# Interleukin-18 deteriorates Fabry cardiomyopathy and contributes to the development of left ventricular hypertrophy in Fabry patients with GLA IVS4+919 G>A mutation

**DOI:** 10.18632/oncotarget.13552

**Published:** 2016-11-24

**Authors:** Yueh Chien, Chian-Shiu Chien, Huai-Chih Chiang, Wei-Lin Huang, Shih-Jie Chou, Wei-Chao Chang, Yuh-Lih Chang, Hsin-Bang Leu, Kuan-Hsuan Chen, Kang-Ling Wang, Ying-Hsiu Lai, Yung-Yang Liu, Kai-Hsi Lu, Hsin-Yang Li, Yen-Jen Sung, Yuh-Jyh Jong, Yann-Jang Chen, Chung-Hsuan Chen, Wen-Chung Yu

**Affiliations:** ^1^ Department of Medical Research, Taipei Veterans General Hospital, Taipei, Taiwan; ^2^ Department of Pharmacy, Taipei Veterans General Hospital, Taipei, Taiwan; ^3^ Division of Cardiology & Department of Medicine, Taipei Veterans General Hospital, Taipei, Taiwan; ^4^ Graduate Institute of Cancer Biology and Center for Molecular Medicine, China Medical University and Department of Biotechnology, Asia University, Taichung, Taiwan; ^5^ Department of Medical Research, Cheng-Hsin Hospital, Taipei, Taiwan; ^6^ College of Biological Science and Technology, National Chiao Tung University, Hsinchu, Taiwan; ^7^ Institute of Pharmacology, Taipei, Taiwan; ^8^ Institute of Clinical Medicine, Taipei, Taiwan; ^9^ Institute of Anatomy and Cell Biology, Taipei, Taiwan; ^10^ Department of Life Sciences and Institute of Genome Sciences, Taipei, Taiwan; ^11^ Genomics Research Center, Academia Sinica, Taipei, Taiwan; ^12^ Department of Chemistry, National Taiwan University, Taipei, Taiwan; ^13^ Faculty of Medicine, National Yang-Ming University, Taipei, Taiwan

**Keywords:** fabry cardiomyopathy, iPSC, enzyme replacement therapy, IL-18

## Abstract

**Rationale:**

A high incidence of GLA IVS4+919 G>A mutation in patients with Fabry disease of the later-onset cardiac phenotype, has been reported in Taiwan. However, suitable biomarkers or potential therapeutic surrogates for Fabry cardiomyopathy (FC) in such patients under enzyme replacement treatment (ERT) remain unknown.

**Objective:**

Using FC patients carrying IVS4+919 G>A mutation, we constructed an induced pluripotent stem cell (iPSC)-based disease model to investigate the pathogenetic biomarkers and potential therapeutic targets in ERT-treated FC.

**Results and methods:**

The iPSC-differentiated cardiomyocytes derived from FC-patients (FC-iPSC-CMs) carried IVS4+919 G>A mutation recapitulating FC characteristics, including low α-galactosidase A enzyme activity, cellular hypertrophy, and massive globotriaosylceramide accumulation. Microarray analysis revealed that interleukin-18 (IL-18), a pleiotropic cytokine involved in various myocardial diseases, was the most highly upregulated marker in FC-iPSC-CMs. Meanwhile, IL-18 levels were found to be significantly elevated in the culture media of FC-iPSC-CMs and patients’ sera. Notably, the serum IL-18 levels were highly paralleled with the progression of left ventricular hypertrophy in Fabry patients receiving ERT. Finally, using FC-iPSC-CMs as *in vitro* FC model, neutralization of IL-18 with specific antibodies combined with ERT synergistically reduced the secretion of IL-18 and the progression of cardiomyocyte hypertrophy in FC-iPSC-CMs.

**Conclusion:**

Our data demonstrated that cardiac IL-18 and circulating IL-18 are involved in the pathogenesis of FC and LVH. IL-18 may be a novel marker for evaluating ERT efficacy, and targeting IL-18 might be a potential adjunctive therapy combined with ERT for the treatment of advanced cardiomyopathy in FC patients with IVS4+919 G>A mutation.

## INTRODUCTION

Fabry disease, resulting from deficiency of α-galactosidase A (α-Gal A) enzyme activity, is an lysosomal storage disorder of glycosphingolipids metabolism and leads to accumulation of glycosphingolipids, particularly globotriaosylceramide in many tissues and cell types [1]. Fabry disease contains several type of mutation at GLA gene loci, and these mutations might cause different effects at GLA, like transcriptional block, low enzyme activity, or functional lost peptide. Cardiac involvement in Fabry disease usually includes left ventricular hypertrophy (LVH), vascular dysfunction, and conduction abnormalities. In Taiwan, a surprisingly high incidence of GLA IVS4+919 G>A mutation of Fabry patients with late-onset cardiac phenotype has been reported. Nevertheless, the natural course and suitable biomarkers for monitoring disease progression remains mostly unclear.

Enzyme replacement therapy (ERT) is currently the only effective therapy to reduce Gb3 accumulations in Fabry disease. Although it improves cardiac function and left ventricular mass, in patients with late phase Fabry cardiomyopathy (FC), ERT introduced a minor reduction in LVH and have not been able to improve myocardial performance [2]. Although Gb3 and globotriaosylsphingosine (lysoGb3) have high sensitivity and correlate with the severity of LVH in FC [3], recent reports have suggested that Gb3 and/or lyoGb3 might not be suitable biomarkers for monitoring the long-term therapeutic outcome of ERT and the progression of FC [4, 5], including that in Fabry patients carrying IVS4+919 G>A mutation [6]. It remains challenging to non-invasively predict the treatment response to ERT. Thus, the identification of pathogenic biomarkers for monitoring patient outcomes in FC, especially in FC patients with myocardial fibrosis, is therefore an urgent need.

Interleukin-18 (IL-18) is a potent pro-hypertrophic inflammatory cytokine and plays a critical role in the pathophysiology of various diseases including myocardial ischemia and myocardial infarction [7]. IL-18 receptor is a heterodimer consisting of a ligand-binding subunit and a signal-transducing subunit. Binding of IL-18 to the ligand-binding subunit recruits the signal-transducing subunit and this activated complex initiates [8] pleiotropic signal transduction events [9, 10]. This cytokine is upregulated under numerous immune, infectious, and inflammatory conditions (11–13). Administration of IL-18 to normal animals results in increased left ventricular mass and a substantial elevation in myocardial collagen content [14, 15]. A direct correlation between IL-18 levels and severity of myocardial dysfunction has been observed [16]. Elevated plasma IL-18 levels have been detected in patients with acute coronary syndromes [17]. In addition, IL-18 levels in both the circulation and resident myocardial tissues are increased in patients with heart failure. IL-18 levels inversely correlated with patient prognosis and lower IL-18 levels were observed in surviving patients [18, 19]. Nevertheless, it remains an open question whether IL-18 plays a role in the pathogenesis underlying FC and LVH.

Induced pluripotent stem cells (iPSCs) provide a feasible platform that can be applied to investigations of tissue repair [20], disease mechanisms [21], drug screening [22] and the regulation of cellular reprogramming [23]. Remarkably, iPSC-derived cardiomyocytes have been considered as a powerful tool for modeling of cardiac diseases [24, 25], assessing of drug cardiotoxicity [26, 27], and investigation of inherited cardiomyopathy [28, 29]. In addition, patient-specific iPSC-derived cardiomyocytes have been used to evaluate the therapeutic efficacy of gene replacement and small molecule inhibitor on lysosomal storage disorders, including Pompe [30] and Fabry disease [31]. Moreover, transcriptomic screening has been used to identify the gene regulatory network in an iPSC-derived cardiac hypertrophy model [32]. These studies indicated that iPSC-derived cardiomyocytes may represent a powerful tool for the investigation of the therapeutic options and biomarkers for FC. In the present study, we isolated blood mononuclear cells from Fabry patients with IVS4+919 G>A mutation and employed patient-specific iPSC-derived cardiomyocytes to model FC and attempted to evaluate the potential biomarker for FC with IVS4+919 G>A mutation. Meanwhile, we also assessed whether neutralization antibody against the identified biomarker could ameliorate the severity of FC in iPSC-derived cardiomyocyte platform. In this present study, we used Affymetrix microarray platform and identified IL-18 as an upregulated factor that may contribute to the pathogenesis of FC and LVH. These findings may help elucidate the underlying mechanisms and identify novel biomarkers for FC and develop novel therapeutic strategies against progressive FC.

## RESULTS

### Establishment of iPSC-derived cardiomyocytes from Fabry patients carrying GLA IVS4+919 G>A mutation

**Figure 1:**
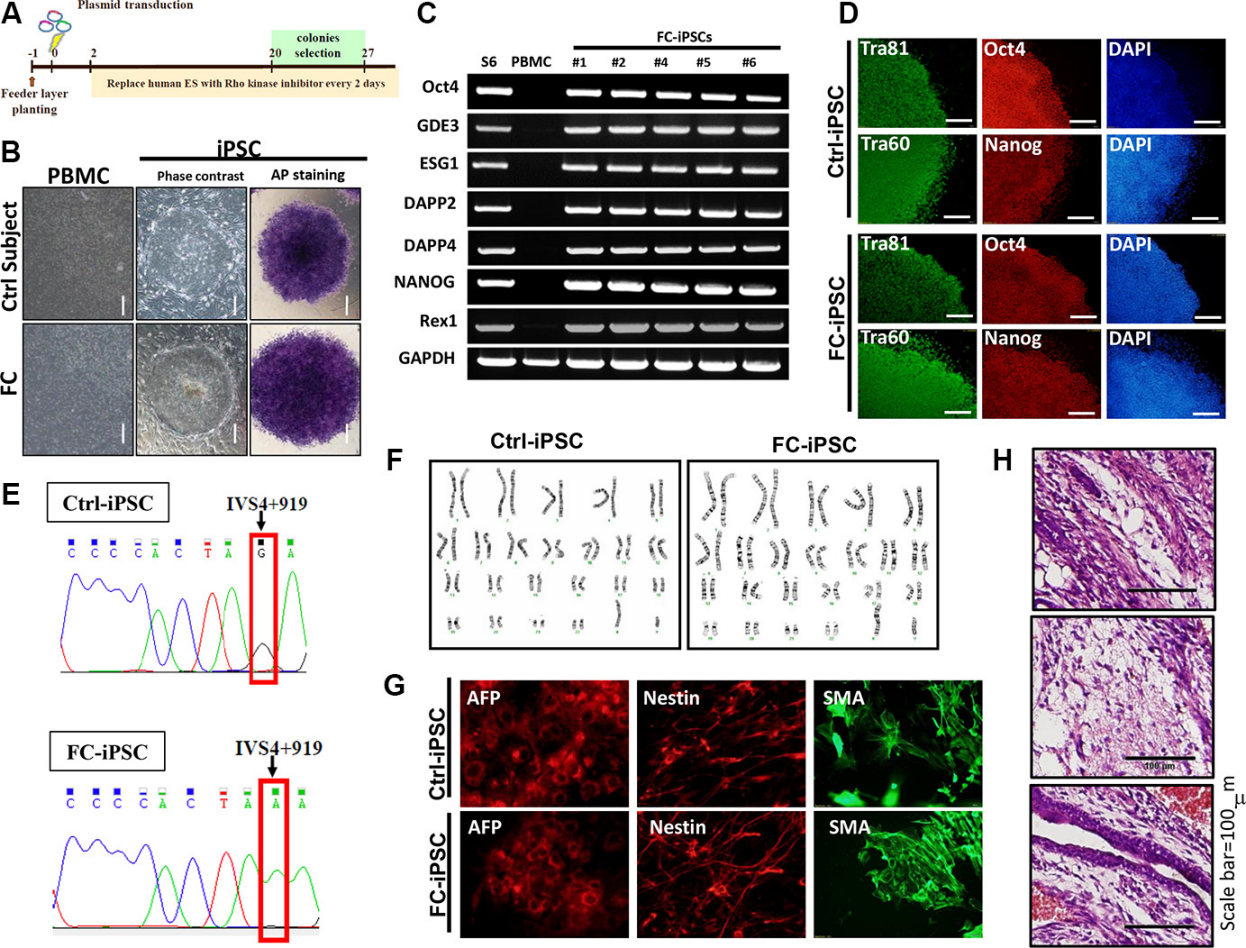
Generation of FC-iPSCs from Fabry patients carrying GLA IVS4+919 G>A mutation (**A**) Protocol for iPSC generation from peripheral blood mononuclear cells (PBMCs) collected from the Fabry cohorts carrying GLA IVS4+919 G>A mutation. (**B**) Phase-contrast photomicrograph and ALP activity in FC-iPSCs. (**C)** Reverse-transcriptase polymerase chain reaction (RT-PCR) results showed that FC-iPSC clones expressed the endogenous pluripotency markers such as OCT4, Nanog, ESG1, DAPP2, DAPP4, REX1, and GDF3. (**D**) Immunofluorescence results indicated that the FC-iPSC and Ctrl-iPSC colonies both showed strong expression of the hESC markers, including Oct4, Nanog, Tra-1-60 and Tra-1-81. (**E**) Sanger sequencing revealed the specific GLA IVS4+919 intron mutation in FC-iPSCs. (**F**) Karyotyping and the abilities for (**G**) teratoma formation and (**H**) *in vitro* tridermal differentiation.

Fabry disease is a genetic lysosomal storage disorder characterized by glycosphingolipid deposition [35, 36]. The cardiac variant of the IVS4 919G>A mutation accounts for 80% of Fabry disease incidence in Taiwan, and FC is one of the major highly prevalent Fabry disease-associated morbidities [37]. The most common presentation of FC is left ventricular hypertrophy (LVH) resulting from the progressive intracellular accumulation of Gb3. In the present study, we attempted to use FC-derived iPSCs to identify and investigate certain candidate factor that contributed to the cardiomyocyte abnormalities associated with GLA IVS4+919 G>A mutation. Ninety-eight Fabry cohorts with the *GLA* IVS4+919 G>A intron mutation were recruited for the study at Taipei Veterans General Hospital between 2010 and 2014. The development of FC was diagnosed by cardiologist. Using electroporation to deliver transcription factors OCT4, SOX2, Lin28, KLF4, and p53 shRNA, peripheral blood mononuclear cells (PBMCs) collected from these cohorts were reprogrammed into patient-specific iPSCs (FC-iPSCs). Control iPSC cell-lines (Ctrl-iPSCs) were simultaneously derived from age-matched health subjects (Figure 1A and 1B). No difference was observed in reprogramming efficiency (data not shown), and the expression of embryonic stem cells marker genes (Tra-1-60 and Tra-1-81) and endogenous pluripotent genes (i.e. OCT4, Nanog, ESG1, DAPP2, DAPP4, REX1, and GDF3) among various patient-derived iPSC lines and Ctrl-iPSCs (Figure 1C and 1D). Using Sanger sequencing, the specific GLA IVS4+919 intron mutation was detected in FC-iPSCs (Figure 1E). Furthermore, these FC-iPSCs also exhibited regular karyotyping, and ability for *in vitro* tridermal differentiation and teratoma formation (Figure 1F–1H).

**Figure 2:**
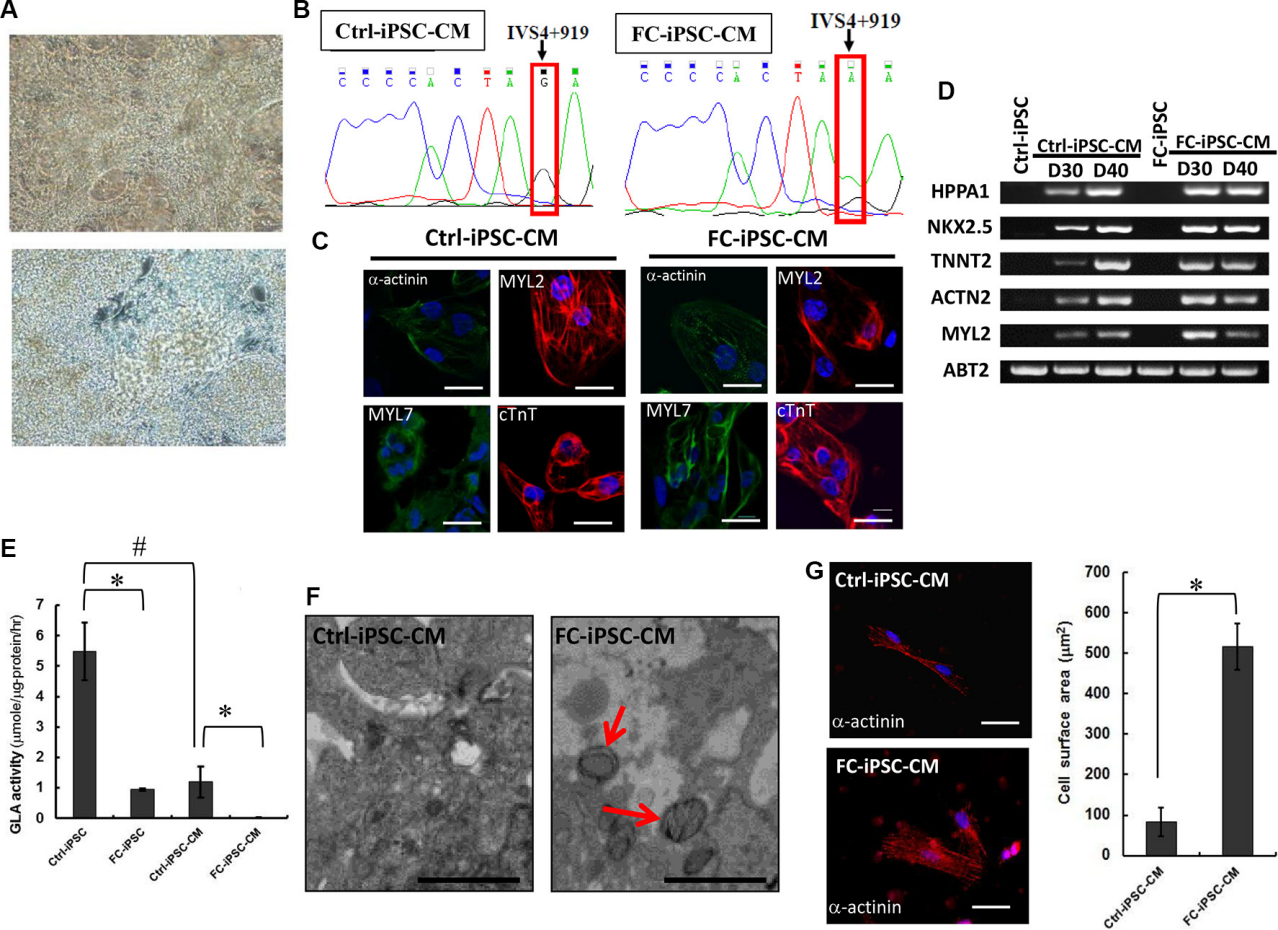
Establishment of FC-iPSC-CMs that recapitulated several FC-specific characteristics (**A**) FC-iPSC-CMs exhibited typical cardiomyocyte morphology and rhythmic contraction at twelve days after cardiac differentiation. (**B**) Sanger sequencing revealed the specific GLA IVS4+919 intron mutation in FC-iPSC-CMs, but not in Ctrl-iPSC-CMs. (**C**) Immunofluorescence results indicated that FC-iPSC-CMs and Ctrl-iPSC-CMs both showed strong expression of myocyte markers, i. e. α-actinin, MYL2, MYL7, and cTnT. (**D**) Reverse-transcriptase polymerase chain reaction (RT-PCR) results showed upregulation of cardiac maker genes (i.e., HPPA1, NKX2.5, TNNT2, ACTN2, and Myl2) in both FC-iPSC-CMs and Ctrl-iPSC-CMs at 30 and 40 days post-induction. (**E**) Measurements ofα-GLA A enzyme activity in Ctrl-iPSCs, FC-iPSCs, Ctrl-iPSC-CMs and FC-iPSC-CMs. (**F**) TEM examination of FC-iPSC-CMs showed the morphological abnormalities (organelle and/or cytoplasm loss) and Gb3 accumulation (appearing within enlarged secondary lysosomes as lamellated membrane structures called zebra bodies) (TEM × 8,000). (**G**) Measurement of cell surface area indicated the cardiomyocyte hypertrophy in FC-iPSC-CMs.

We next employed conventional cardiomyocyte differentiation protocol and differentiated these FC-iPSCs into cardiomyocytes (FC-iPSC-CMs). Both FC-iPSC-CMs and Ctrl-iPSC-CMs exhibited typical cardiomyocyte morphology and rhythmic contraction at twelve days after cardiac differentiation (Figure 2A). We further used Sanger sequencing and confirmed the GLA IVS4+919 intron mutations in FC-iPSC-CMs, but not Ctrl-iPSC-CMs (Figure 2B). Immunofluorescence indicated that, several distinct myocyte markers, i.e. α-actinin, MYL2, MYL7, and cTnT were extensively stained in FC-iPSC-CMs and the Ctrl-iPSC-CMs at 30 days post-induction (Figure 2C). RT-PCR also revealed the upregulation of cardiac maker genes (i.e., HPPA1, NKX2.5, TNNT2, ACTN2, and Myl2) in both FC-iPSC-CMs and Ctrl-iPSC-CMs at 30 and 40 days post-induction (Figure 2D). Notably, no significant discrepancies in the efficiency of cardiac differentiation as well as in the expression levels of these cardiomyocytes markers were observed between these two cells. We further examined whether these FC-iPSC-CMs also exhibited FC-specific characteristics after cardiac differentiation. At post-differentiation 40 days in Ctrl and FC-derived cells, α-GLA A enzyme activity in the differentiated cardiomyocytes were significantly higher than that in iPSCs. Remarkably, α-GLA A enzyme activity were substantially reduced in FC-iPSCs and FC-iPSC-CMs, compared with their corresponding Ctrl cells (Figure 2E). Lysosomal abnormalities and Gb3 accumulation were observed in FC-iPSCs-CMs but not Ctrl-iPSCs-CMs at 40 days post-induction, and the FC-iPSC-CMs from all twelve patients displayed similar cardiac hypertrophy and TEM patterns (Figure 2F). In addition, FC-iPSC-CMs exhibited 6-fold higher surface area than that in Ctrl-iPSC-CMs (Figure 2G), revealing the typical phenotypes of cardiomyocyte hypertrophy. Taken together, these FC-iPSC-CMs with IVS4G>A mutation recapitulated several FC-specific phenotypes including lysosomal Gb3 accumulation, cellular hypertrophy and reduced α-GLA A enzyme activity. This FC-iPSC-CM may represent ideal *in vitro* platform for investigating the pathogenesis of FC and therapeutic strategy for FC.

### Identifying IL-18 as an abundant factor in hypertrophic FC-iPSC-CMs

**Figure 3:**
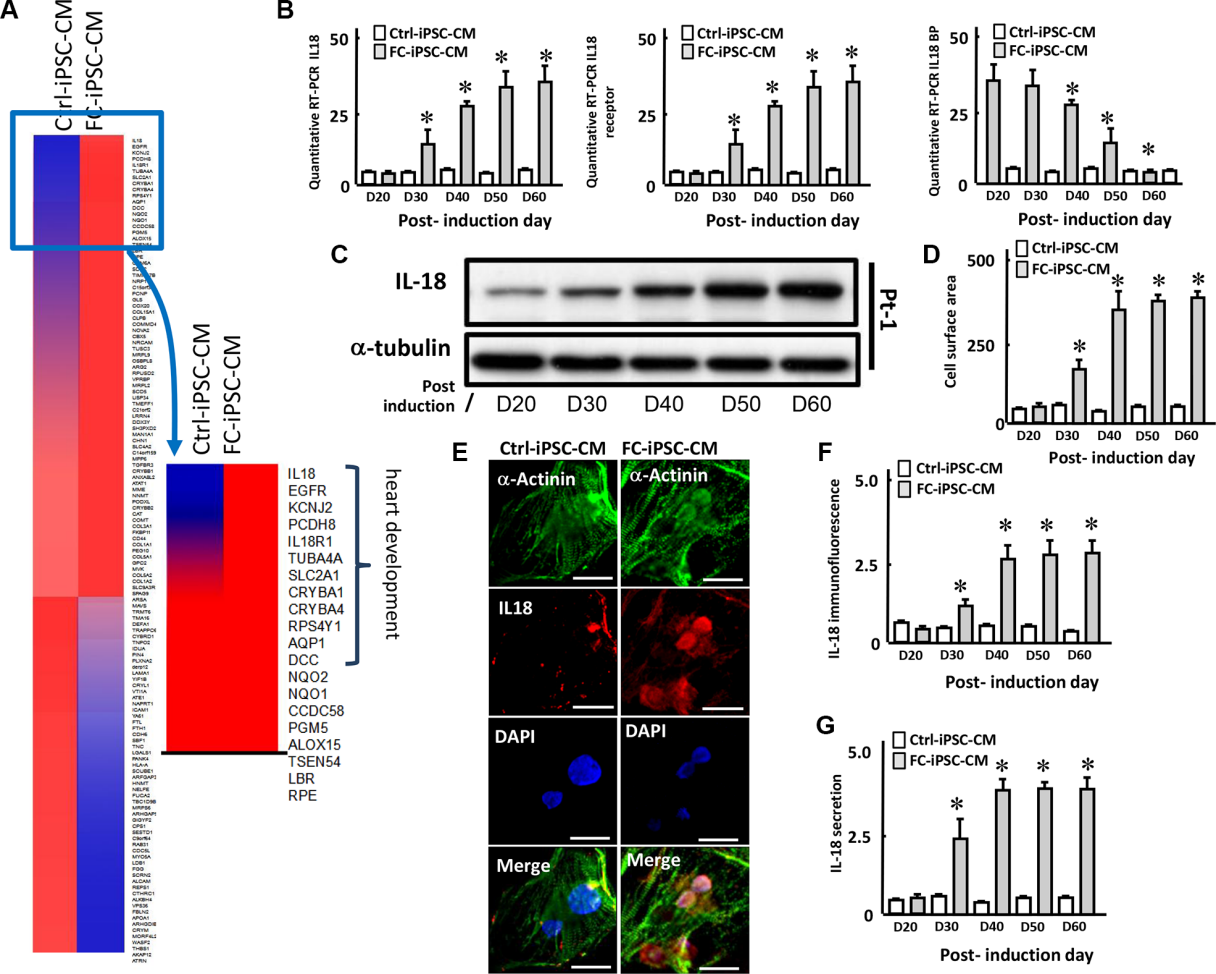
L-18 as a highly upregulated factor in FC-iPSC-CMs (**A**) Heatmap depicting fold change of expression in the different genes between FC-iPSC-CM and iPSC-CM. Most different genes between two cells expression are grouped in heart development. (**B**) Quantitative RT-PCR revealed the expression of IL-18, IL-18 receptor (IL-18R) and IL-18 binding protein (IL-18BP) along the differentiation course. (**C**) Western blot showed the expression of IL-18 during cardiomyocyte differentiation. (**D**) Measurement of cell surface area in FC-iPSC-CMs during the cardiomyocyte differentiation. (**E**) Immunofluorescence result and (**F**) the quantification indicated the IL-18 upregulation in FC-iPSC-CMs than that in Ctrl-iPSC-CMs at post-induction 40 day. (**G**) ELISA showed the secretion of IL-18 into the conditioned media during the cardiac induction.

To identify the most abundant factor differentially expressed between Ctrl-iPSC-CMs and FC-iPSC-CMs, we used Affymetrix microarray platform to identify the differentially expressed genes (Figure 3A). After 40 days of cardiac differentiation, the RNA of FC-iPSC-CMs and Ctrl-iPSC-CMs were extracted, and then subjected to quality verification by Agilent 2100 Bioanalyzer. Following RNA quality verification, the cDNA were synthesized, labeled, and hybridized with Affymetrix human gene chip. The results depicted in the heatmap of Figure 3A showed that a subset of genes is expressed at moderate levels in Ctrl-iPSC-CM with a trend toward higher expression in FC-iPSC-CMs. These genes included the markers and function of heart development likes IL-18, EGFR, KCNJ2, PCDH8 and IL-18R1. In addition, the other genes associated with heart development, such as TUBA4A, SLC2A1, CRYBA1 and CRYBA4 also exhibited higher expression levels in FC-iPSC-CMs. (Figure 3A). Among these subsets of genes that were highly expressed in FC-iPSC-CMs, IL-18 was the most upregulated genes expressed in FC-iPSC-CMs, raising the possibility that interleukin-18 (IL-18) may act as a potential biomaker in FC-associated cardiomyopathy.

We next evaluated the regulation of IL-18 expression at mRNA and protein levels in FC-iPSC-CMs during the differentiation course. Quantitative RT-PCR revealed that IL-18 and its receptor (IL-18R) were both upregulated, whereas IL-18 binding protein (IL-18BP) was downregualted, in a time-dependent manner along the differentiation course (Figure 3B). Western blot revealed that IL-18 was initially increased at post-induction Day 30, and was further upregulated time-dependently and reached plateau at post-induction Day 50 (Figure 3C). By measuring the cell surface area, a severe cardiomyocyte hypertrophy was detected at post-induction 40 day in FC-iPSC-CMs (Figure 3D). In addition, immunofluorescence also indicated that IL-18 protein was significantly higher in FC-iPSC-CMs than that in Ctrl-iPSC-CMs at post-induction 40 day (Figure 3E and 3F). Furthermore, ELISA indicated that IL-18 was secreted into the conditioned media and elevated in the manner identical to IL-18 protein during the cardiac induction (Figure 3G). These findings revealed that IL-18 is a FC-specific factors upregulated during the differentiation process in FC-iPSC-CMs.

### IL-18 as a candidate FC biomarker in FC patients with IVS4G>A mutation

**Figure 4:**
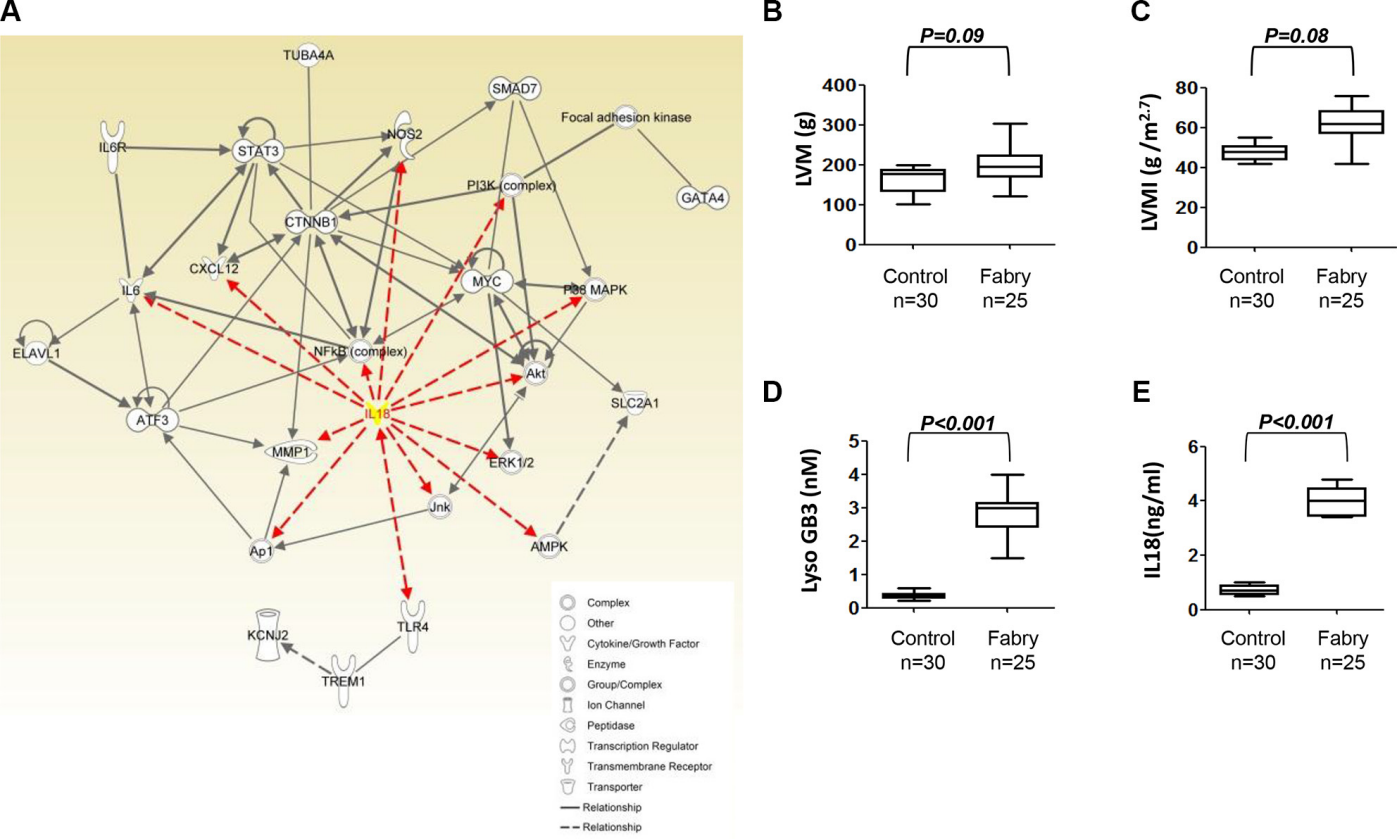
Upregulation of IL-18 in Fabry patients carrying GLA IVS4+919 intron mutation (**A**) The associated interaction network was defined by Ingenuity Pathway Analysis (IPA) for IL-18 and the cardiac hypertrophy related genes. The IL-18 was labeled as yellow and the interaction linage between IL-18 and other molecules was plotted as red-line. (**B**, **C**) Transthoracic echocardiography parameters and the serum levels of (**D**) lysoGb3, and (**E**) IL-18 in 25 FC patients carrying GLA IVS4+919 intron mutation and without the development of LVH.

In order to determine the key molecules that involved in the FC, the Ingenuity Pathway Analysis (IPA) was used to address the interaction network of the differentially expressed genes in FC-iPSC-CMs. Mapping these genes to the network of cardiac hypertrophy revealed the vital role of IL-18 that participate in FC (Figure 4A). IL-18 was illustrated to interact with the cytokines/chemokines (IL6, and CXCL12), cytosolic signaling molecules (NOS2, PI3K/Akt, MAPK cascade, and AMPK), and redox transcription factors (AP1, and NF-κB). Moreover, the IL-18-signaling downstream effectors, i.e. ATF3, CTNNB1, STAT3, SMAD7, GATA4, focal adhesion kinase, and MMP1 have also been illustrated by IPA. All of these molecules play the important role in the progression of hypertrophic cardiomyopathy. Overall, the bioinformatics approach using IPA markedly pointed out that IL-18 acts as a critical initiator in the network of cardiac hypertrophy, suggesting the potential involvement of the secreted IL-18 on FC progression.

To further elucidate the role of IL-18, we also assessed whether IL-18 is also elevated in the circulation in clinical samples from Fabry patients. First, we examined the circulatory IL-18 levels in 25 Fabry patients who carried IVS4+919 G>A mutation and have not developed left ventricular hypertrophy (LVH). There was no significant difference in transthoracic echocardiography parameters including left ventricular mass (LVM) and left ventricular mass index (LVMI) (all transthoracic echocardiography parameters *P* > 0.05, Figure 4B and 4C). Notably, these patients exhibited slightly higher elevation in conventional FC-specific biomarker lysoGb3, as well as IL-18 (Figure 4D and 4E).

**Figure 5:**
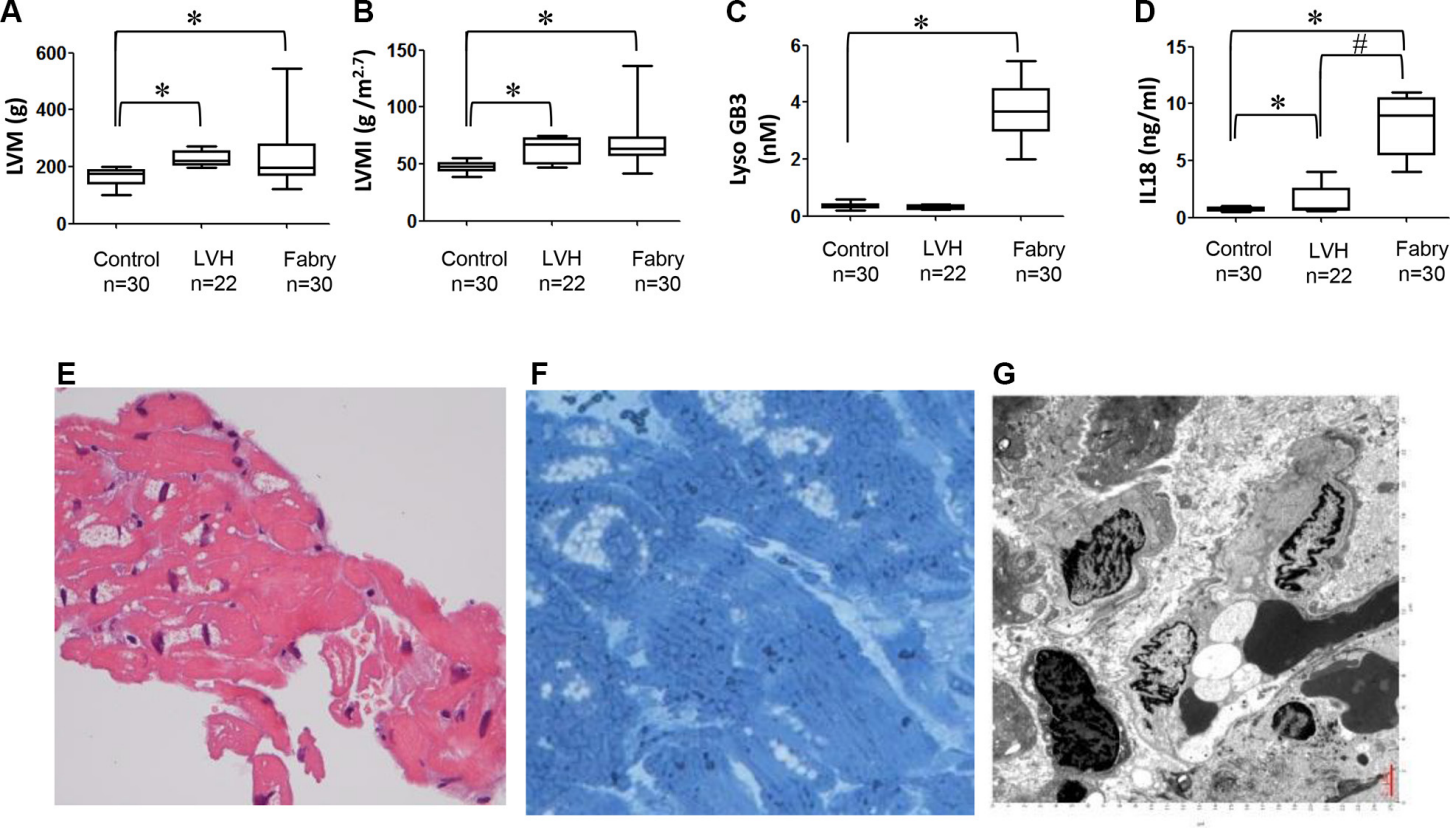
Comparison of secretory IL-18, LysoGb3 and transthoracic parameters among FC patients with LVH, hypertensive patients with LVH only and control subjects (**A**–**D**) Transthoracic echocardiography parameters and the serum levels of lysoGb3, IL-18 in 30 FC patients with LVH, 22 hypertensive patients with LVH only, and 30 control subjects. (**E**) Histologic examination (H&E) staining of the myocardium showed markedly hypertrophic and disorganized myocytes with large perinuclear and sarcoplasmic vacuoles (upper right). (**F**) Toluidine blue staining of the myocardium verified the accumulation of glycosphingolipids (lower left). The scale bar is 250 μm. (**G**–**H**) Transmission electron microscopic examination of the myocardium revealed lamellar bodies (zebra bodies) representing lysosomes containing glycolipids, × 60,000 (lower right).

Next, we recruited 30 Fabry patients with IVS4+919 G>A mutation and have never received enzyme replacement therapy and have developed Fabry cardiomyopathy (FC), and further compared their transthoracic echocardiography and serum levels of LysoGb3 and IL-18, with those in age-matched control subjects or hypertensive patients with LVH only (Table 1). The severity of LVH in these patients was diagnosed by transthoracic echocardiography. Comparing with those parameters in age-matched control subjects, both Fabry patients with IVS4+919 G>A mutation and patients with LVH exhibited high magnitudes of LVM and LVMI (all transthoracic echocardiography parameters *P* < 0.001, Figure 5A and 5B). In addition to all echocardiography parameters, the serum lysoGb3 concentration was higher in Fabry patients with IVS4+919 G>A mutation, but not in patients with LVH alone, comparing with the controls (Figure 5C). Remarkably, the serum IL-18 concentrations were slightly increased in patients with LVH alone, and were even much higher in Fabry patients carried IVS4+919 G>A mutation and have developed LVH (Figure 5C and 5D). Histological examination and transmission electron microscopic examination were used to validate the microscopic characteristics in these Fabry patients who have developed FC. A hematoxylin and eosin examination of the biopsied myocardium sample revealed cardiomyocyte hypertrophy and disorganization with large perinuclear and sarcoplasmic vacuoles (Figure 5E). Toluidine blue staining of the biopsied myocardium indicated the accumulation of glycosphingolipids (Figure 5F). Transmission electron microscopic examination of the myocardium revealed the formation of lamellar bodies (zebra bodies) which represent lysosomes containing glycolipids (Figure 5G and 5H). Collectively, these *in vitro* and clinical findings (Figures 3, 4, 5) indicated that IL-18 is highly elevated and associated with Fabry patients with FC or LVH and could be used as a diagnostic marker in Fabry patients who have developed LVH.

**Table 1:** Patient information

	Healthy control *N* = 30	LVH *N* = 22	Fabrycardiomyopathy (FC) *N* = 25
Age, yrs	59.1 ± 4.5	55.2 ± 6.2	58.4 ± 5.8
Women, no. (%)	1 (3%)	2 (9%)	1 (4%)
High, cm	155 ± 5.3	157 ± 4.2	156 ± 3.6
Weight, kg	56 ± 5.5	57 ± 5.8	56 ± 3.9
Body mass index, kg/m2	26.1 ± 2.2	25.4 ± 2.4	25.8 ± 2.9
MSSI cardiovascular score	-	-	12.1 ± 2.2
Hypertension, no. (%)	-	12 (54%)	12 (48%)
LVH, no. (%)	-	22 (100%)	25 (100%)
Ccr < 60 ml/min, no. (%)	-	22 (100%)	25 (100%)
ACE/ARB inhibitors use	-	22 (100%)	20 (80%)
Left ventricular mass, g	169 ±7.3	232.3 ±20.5	260.4 ± 23.1
Left ventricular mass index	50 ± 0.6	65.1 ± 2.5	71.5 ± 3.5
Left ventricular ejection fraction, %	70%	65%	69%
NT-pro-BNP	-	984± 10.5	703± 12.5
LysoGb3, nM	0.3 ±0.01	0.3 ±0.03	3.5 ± 0.4
IL18, ng/mL	0.8 ±0.01	0.5 ±0.04	7.5 ± 0.2

Data are mean ± SD. MSSI, Mainz severity score index; Ccr: Creatinine Clearance.

### Enzyme replacement therapy decreased left ventricular hypertrophy and ameliorated serum levels of lysoGb3 and IL-18 in patients with fabry cardiomyopathy

**Figure 6:**
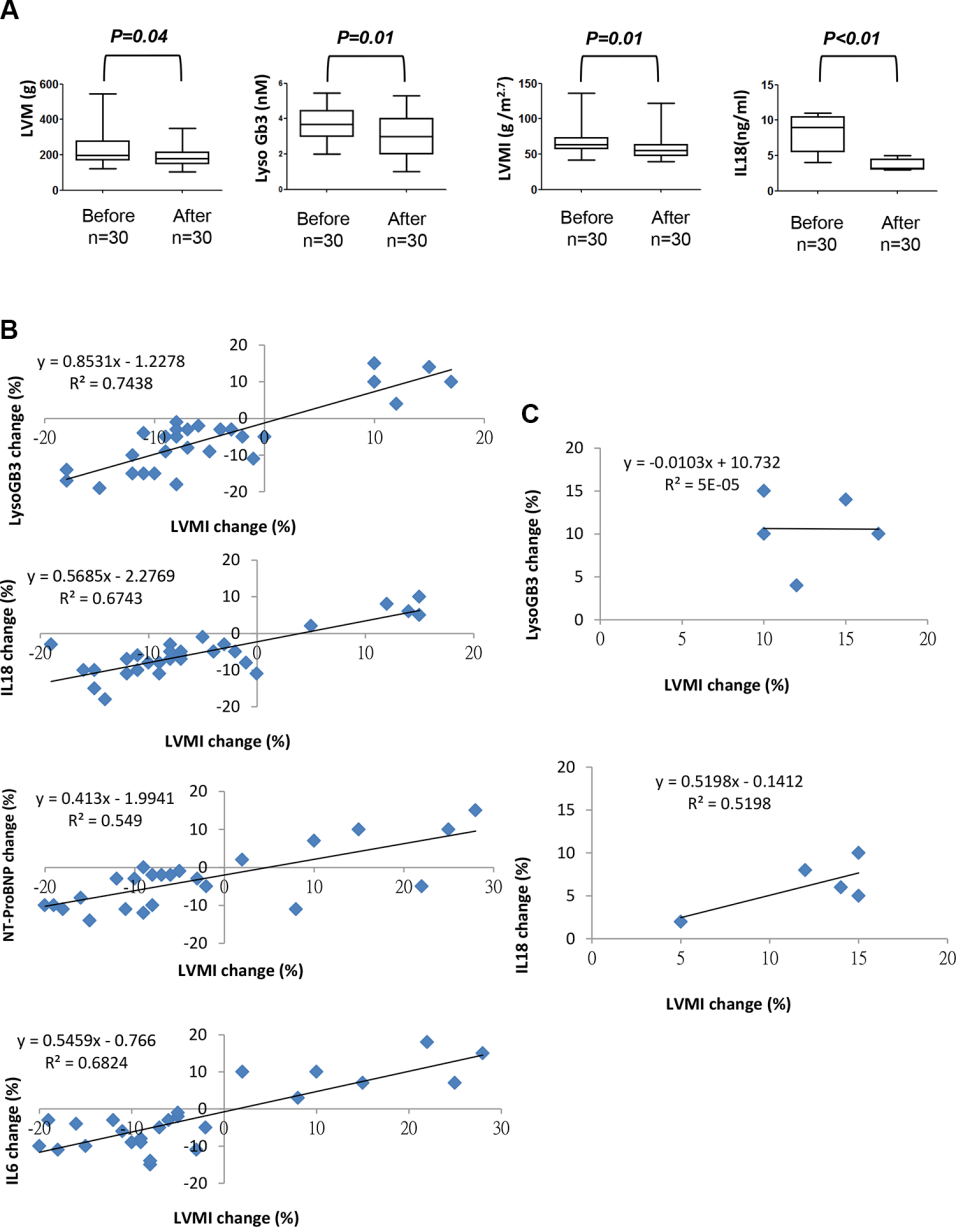
Comparison of secretory IL-18, LysoGb3 and LVH severity in FC patients before and after ERT treatment (**A**) Transthoracic echocardiography parameters and lysoGb3 and IL-18 levels in 30 FC patients before and after ERT. (**B**) The linear regression between changes in the left ventricular mass index and changes in the levels of the biomarkers in all 30 FC patients with ERT and (**C**) in 5 FC patients with LVH progression.

**Figure 7:**
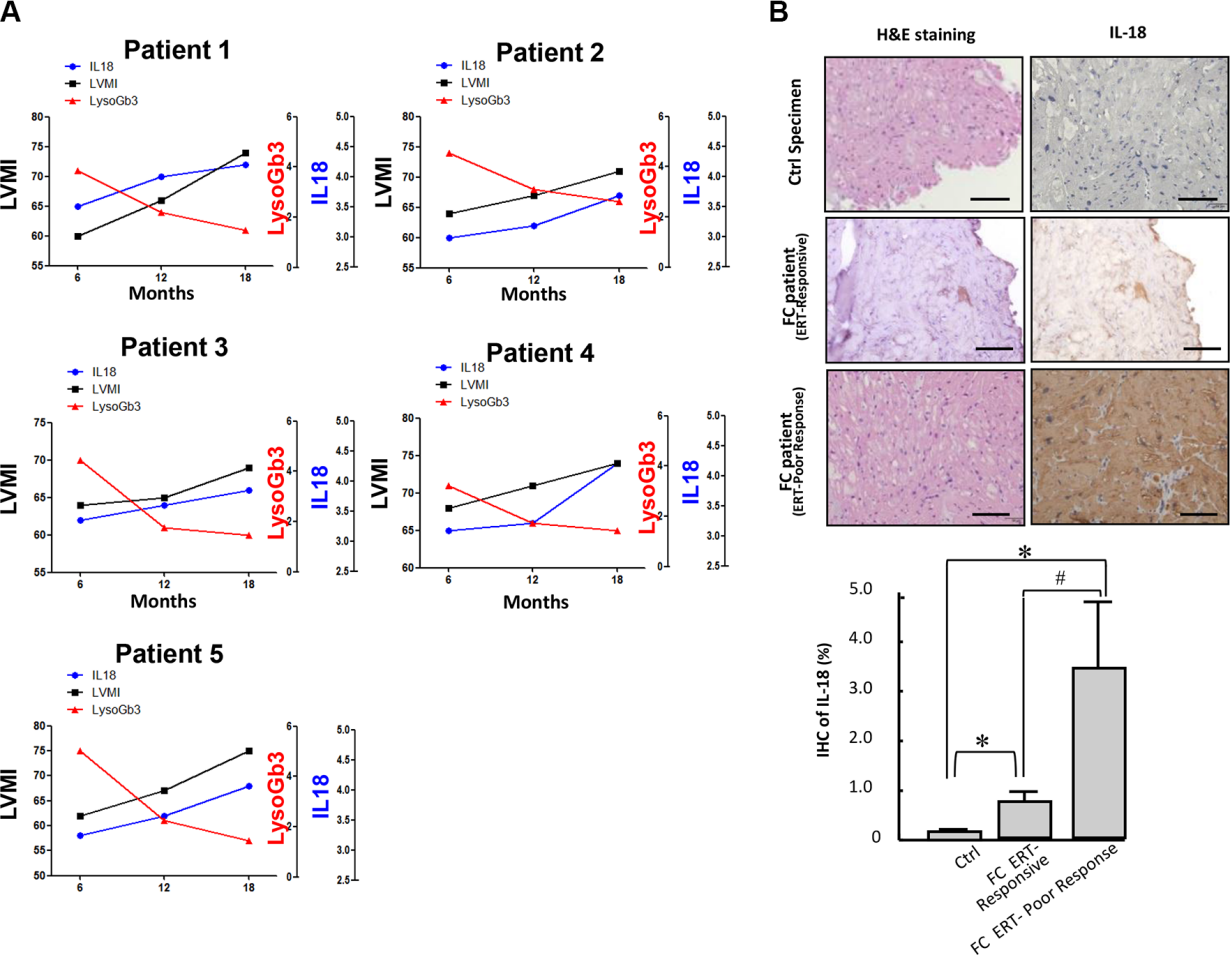
Serum levels and cardiac expression pattern of IL-18 in FC patients with poor response to ERT (**A**) The changes of levels of IL-18 and LysoGb3 and the progression of LVH in 5 FC patients with poor response to ERT. (**B**) Immunohistochemistry indicated IL-18 upregulation in myocardial biopsy samples from FC patients with poor responses to ERT compared with those who responded well to ERT.

Because our data indicated that the serum levels of IL-18 were significantly upregulated in patients diagnosed as FC, we evaluated whether IL-18 could serve as circulating biomarkers that mirror ERT efficacy and disease progression in FC. A total of 30 Fabry patients carried IVS4+919 G>A mutation and have developed LVH and received ERT were enrolled in this study, and LVH reduction was used as a surrogate endpoint to compare several parameters before and after ERT. The severity of LVH, as indicated by echocardiography parameters and lyoGb3 concentration, was reduced after ERT ((Δ = −38.7 (*P* = 0.04), −9.7 (*P* = 0.01), and −2.1 (*P* = 0.01), for LVM, LVMI, and lyoGb3, respectively) along with the reduction of IL-18 (Δ = −4.5 (*P* < 0.01)) (Figure 6A). We previously have reported that pro-inflammatory cytokines, particularly IL-6 and MCP-1, were elevated in patients with FC and carrying GLA IVS4+919 G>A mutation [38]. In addition, N-terminal pro-brain natriuretic peptide is a marker of left ventricular hypertrophy in hypertrophic cardiomyopathy [39]. Subsequently, we evaluated the correlation between the changes in nt-ProBNP, lysoGb3, IL-6, IL-18 and the changes in LVMI. The correlations between LVMI and these serum biomarkers were all positive (beta = 0.55 (*P* < 0.05), 0.68 (*P* < 0.05), 0.74 (*P* < 0.05), and 0.67 (*P* < 0.05) for nt-ProBNP, IL-6, lyoGb3 and IL-18, respectively) (Figure 6B). The beta values for IL-18 and IL-6 were equivalent, whereas considering the critical roles of IL-18 in heart diseases and cardiac hypertrophy, IL-18 may represent a better biomarker that can reflect the development of FC in patients with IVS4+919 G>A mutation. We further assessed the correlation between the changes in IL-18 or LysoGb3 and the changes in LVMI in 5 of 30 patients with LVH progression after ERT. Only IL-18 was positively correlated with LVMI (beta = 0.51 (*P* < 0.05), respectively), whereas lyoGb3 had no correlation with LVMI (beta = 5 × 10^−5^) (Figure 6C). This discordance between reduced lysoGb3 and the progression of LVH signifies its limitation for prognostic implications. Meanwhile, the levels of IL-18 were elevated and highly paralleled the progression of LVH even under standard ERT (Figure 7A). Finally, immunohistochemical results further confirmed that the expression levels of IL-18 were significantly increased in the biopsy samples of FC patients with poor responses to ERT compared with those who responded well to ERT (Figure 7B). Collectively, our clinical findings revealed that conventional marker lysoGb3 may not be a reliable marker for Fabry-associated ventricular hypertrophy, particularly in patients with LVH progression after ERT. Alternatively, serum IL-18 level may be an ideal biomarker for revealing Fabry-associated cardiac manifestations with high sensitivity, specifically for monitoring progressive LVH in FC patients undergoing ERT.

### Neutralization of IL-18 enhances ERT efficacy on cardiomyocyte hypertrophy in FC-iPSC-CMs

Disease-specific iPSCs provide a high-throughput platform for personalized drug screening and clinical-oriented therapeutic development [40]. Therefore, we evaluated the treatment response of FC-iPSC-CMs to ERT. Six FC-iPSC-CM clones that consistently exhibited Fabry-associated characteristics, including cardiomyocyte hypertrophy, decreased GLA expression/activity, and lysosomal abnormalities at 40–60 days post-induction, were allocated for subsequent experiments.

**Figure 8:**
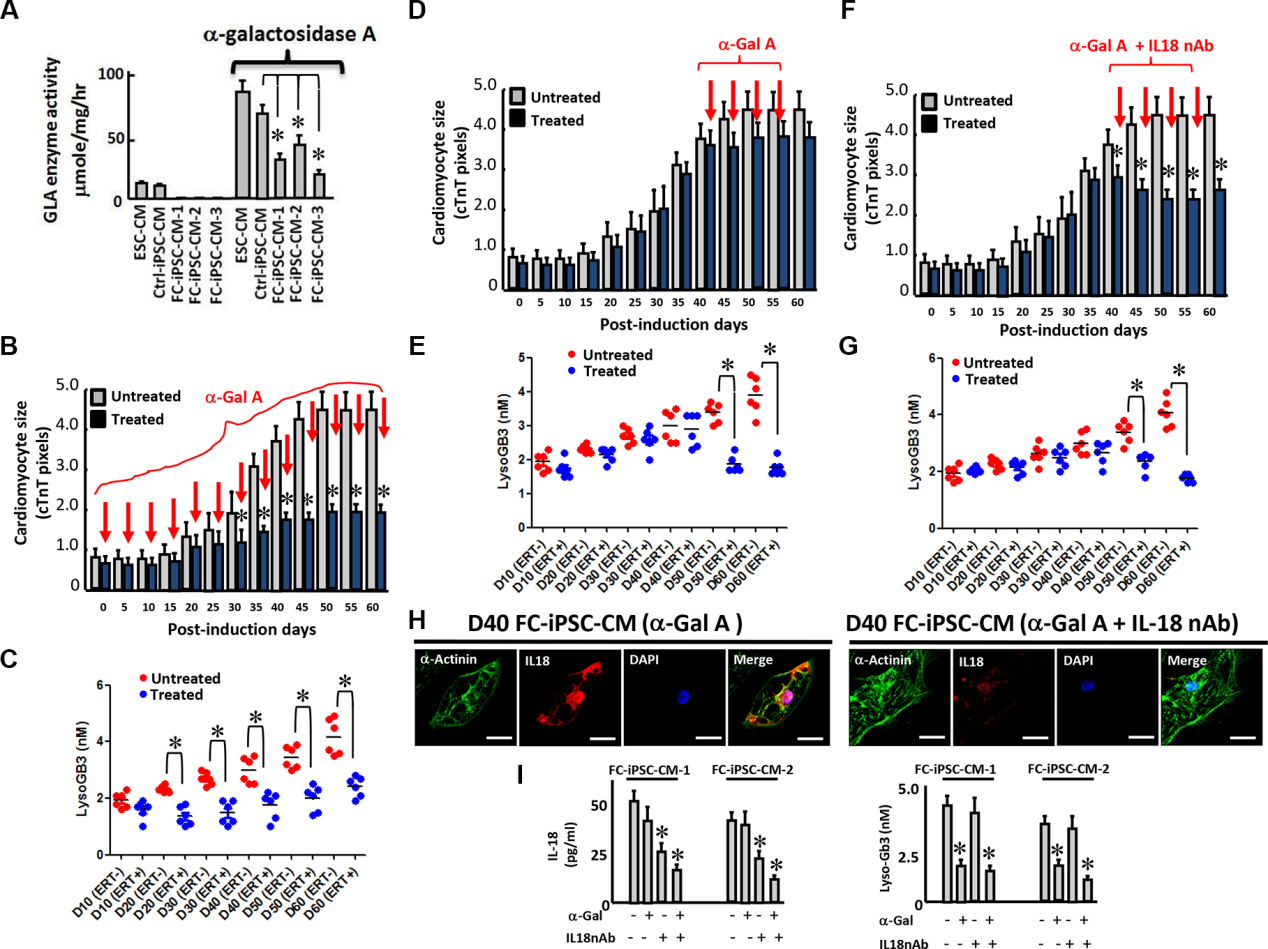
A combination of the IL-18 neutralization and ERT enhances the efficacy of ERT in FC-iPSC-CMs (**A**) Increased GLA activity in ESC-CMs, Ctrl-iPSC-CMs and various clones of FC-iPSC-CM after the administration of alpha-galactosidase A. (**B**) The effect of alpha-galactosidase A administration at the onset of the cardiac induction period on cardiomyocyte size. (**C**) The effect of alpha-galactosidase A administration at the onset of the cardiac induction period on lysoGb3 secretion into the culture medium. (**D**) A protocol for the combination therapy of IL-18 neutralization and alpha-galactosidase A in FC-iPSC-CMs. (D) The effect of alpha-galactosidase A administration on cardiomyocyte size at day 40 of the cardiac induction period. (**E**) The effect of alpha-galactosidase A administration on lysoGb3 secretion into the culture medium at day 40 of the cardiac induction period. (**F**) The effect of the combination of alpha-galactosidase A plus IL-18 neutralization on cardiomyocyte size at day 40 of the cardiac induction period. (**G**) The effect of the combination of alpha-galactosidase A plus IL-18 neutralization on lysoGb3 secretion into the culture medium at day 40 of the cardiac induction period. (**H**) Immunofluorescence indicating the effects of alpha-galactosidase A alone and the combination of the two agents on the expression of IL-18 in the FC-iPSC-CM platform. Scale bar = 10 μm. (**I**) ELISA indicating the effects of alpha-galactosidase A alone and the combination of the two agents on the production of IL-18 and LysoGb3 from the FC-iPSC-CM platform.

First, we tested whether ERT could restore *in vitro* GLA activity in FC-iPSC-CMs at 30 days post-induction. The administration of alpha-galactosidase A (Replagal 5 μg/ml for 4 days) elicited high GLA activity in ESC-derived cardiomyocytes, Ctrl-iPSC-CMs and FC-iPSC-CMs. Notably FC-iPSC-CMs exhibited a significantly lower GLA activity than the control cells after alpha-galactosidase A administration (Figure 8A).

Second, we tested the treatment response of alpha-galactosidase A at early onset on cardiomyocyte hypertrophy in FC-iPSC-CMs. To ensure the restoration of GLA activity, medium containing alpha-galactosidase A was refreshed every two days. There was a significant decrease in cardiomyocyte size on day 35 in the FC-iPSC-CMs that were treated from post-induction day 0 to day 60 (Figure 8B). At day 60, the cell size of the treated FC-iPSC-CMs was approximately 31% smaller than that of the untreated FC-iPSC-CMs (Figure 8B). LysoGb3 release was also reduced by alpha-galactosidase A, which is a known effect of this enzyme, during the differentiation course of the FC-iPSC-CMs (Figure 8C). These data showed that ERT effectively prevented the development of cardiomyocyte hypertrophy in early onset FC.

Third, we attempted to test the efficacy of ERT in FC-iPSC-CMs with severe cardiomyocyte hypertrophy at post-induction day 40 (Figure 8D and 8E). Alpha-galactosidase A treatment from post-induction day 40 to day 60 resulted in a negligible inhibitory effect on cardiomyocyte size (Figure 8D), despite reduced levels of lysoGb3 secretion (Figure 8E). Meanwhile, we examined whether IL-18 could serve as therapeutic targets at this stage, and we also tested the treatment efficacy of the neutralization of IL-18 in combination with ERT (Figure 8F and 8G). Remarkably, the addition of an IL-18 neutralization antibody (IL-18 nAb) potently enhanced the efficacy of alpha-galactosidase A and reduced the cardiomyocyte size (Figure 8F), accompanied by a decrease in the production of lysoGb3 (Figure 8G). Immunofluorescence further indicated that the cardiac-specific marker α-actinin was unaffected, whereas fewer FC-iPSC-CMs expressed IL-18 when receiving the combination treatment than the cells receiving ERT only (Figure 8H). As detected by ELISA, the secretion of IL-18 (Figure 8I), were generally unaffected by ERT alone but were moderately attenuated by IL-18 nAb (Figure 8I, left). Similarly, the addition of IL-18 nAb enhanced the effects of ERT and exhibited a synergistic efficacy that potently suppressed IL-18 (Figure 8I, left). The release of lysoGb3 was consistently reduced by administration of Alpha-galactosidase A but not IL-18 nAb (Figure 8I right).

Furthermore, we chose several cardiac hypertrophy-associated genes (i.e. ANF, ACTC1, MYL2, and MYL7) that are upregulated in FC-iPSC-CMs (unpublished data) and examined the efficacy of alpha-galactosidase A (Replagal), IL-18 nAb, or alpha-galactosidase A plus IL-18nAb on the expression of these hypertrophy-associated genes. As detected by quantitative RT-PCR, alpha-galactosidase A alone did not significantly affect these genes. IL-18 nAb alone showed a moderate suppressive effect and the combination of alpha-galactosidase A plus IL-18 nAb synergistically suppressed these selected hypertrophy-associated genes (Supplemental Figure S1). These data indicated the ineffectiveness of ERT drug on the expression of cardiac hypertrophy-associated genes as well as cardiomyocyte hypertrophy. These *in vitro* findings using FC-iPSC-CMs were consistent with clinical observations by Kampmann et al. and our previous study [41, 42].

### Low GLA expression might not be the predominant factor that stimulates the IL-18 secretion

**Figure 9:**
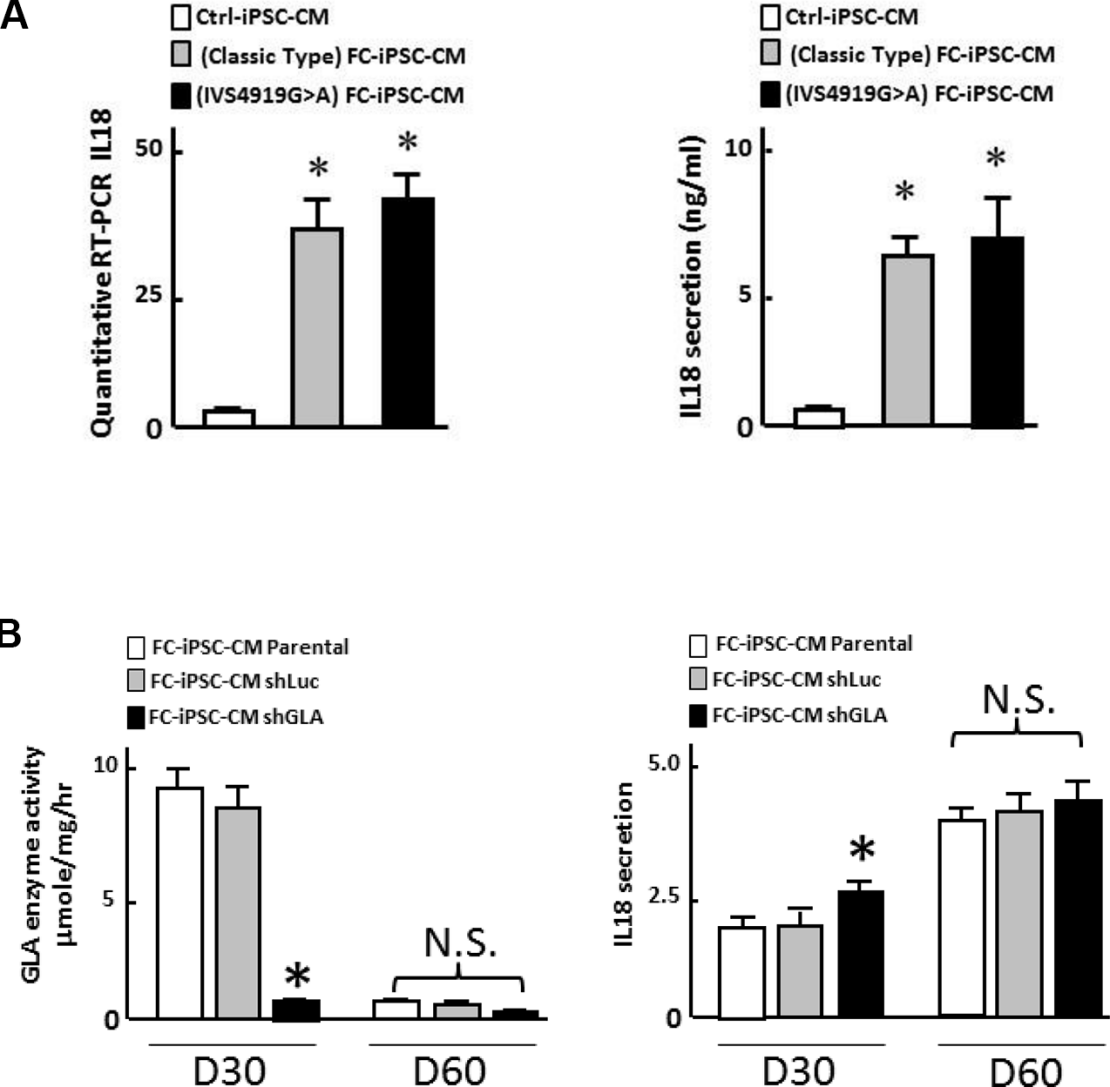
Evaluation of the interrelationship of GLA activity and IL-18 secretion (**A**) Comparison of GLA activity and IL-18 secretion patterns in FC-iPSC-CMs derived from Fabry cohorts with GLA IVS4+919 G>A mutation or classic type mutation. (**B**) Effect of GLA knockdown using shRNA against GLA on GLA activity and IL-18 at post-induction 30 days and 60 days. Data shown here is the mean ± SD of three independent experiments, *P < 0.05 vs. parental and ShLuc. These experiments were conducted in FC-iPSC-CM derived from two individual FC patients.

In addition to the enrolled Fabry patients with the IVS4+919 G>A mutation predominantly found in Taiwan, we have also recruited a small population of Fabry cohorts with the classic type mutation. To examine the IL-18 expression pattern, we also collected the peripheral mononuclear blood cells from Fabry patients with classic type mutation and reprogrammed them into FC-iPSC (classic type). Subsequently, we also differentiated them into FC-iPSC-CMs (classic type), and further compared the expression and secretion patterns of IL-18 in FC-iPSC-CMs (classic type) with that in FC-iPSC-CMs (IVS4+919 G>A) and Ctrl-iPSC-CMs. A moderate IL-18 upregulation as well as its secretion were observed in FC-iPSC-CMs (classic type). These data related to classic type mutation are showed in the Figure 9A.

To assess the consequence of GLA knockdown on IL-18 secretion, we compared the IL-18 secretion and GLA activity in FC-iPSC-CMs receiving shRNA against GLA (shGLA) or shLuc at different time points after cardiac induction (D30, and D60). Knockdown of GLA by ShGLA led to expected reduction in GLA activity in FC-iPSC-CMs at post-induction 30 days, compared with the same cells receiving ShLuc or those without any treatment. Nevertheless, no difference in GLA activity was detected in FC-iPSC-CMs among untreated and shGLA-treated cells at post-induction 60 days. Remarkably, a significant increase in IL-18 secretion was observed in FC-iPSC-CMs with GLA knockdown at post-induction day 30, whereas high levels of IL-18 secretion were detected in FC-iPSC-CMs among all groups with any given treatment (Figure 9B). Our *in vitro* data using Fabry patient-derived iPSC platform indicated that low GLA expression only moderately elicited IL-18 secretion, suggesting that low GLA expression might not be the predominant factor that stimulates the IL-18 secretion.

## DISCUSSION

Gal A enzyme deficiency in Fabry patients results in progressive accumulation of cellular Gb3 in a variety of cell types, including the walls of small blood vessels, tubular epithelial cells, nerves, dorsal root ganglia, renal glomerular and cardiomyocytes [43, 44]. The GLA IVS4+919 G>A mutation has been revealed as a high incidence of GLA mutation (∼1 in 1,500–1,600 males) of Fabry patients in Taiwan [45]. Moreover, this mutation has been reported in patients with the late-onset cardiac phenotype, particular the left ventricular hypertrophy [46, 47]. However, due to the limitations of clinical samples from cardiac biopsies or primary cardiomyocyte cultures, the investigations of the pathogenesis of FC are largely hindered. Although a variety of mutations of the α-Gal A gene have been identified to cause Fabry disease (Human Gene Mutation Database), the pathogenetic mechanism leading to FC remains blurred. In Taiwan Fabry population with high incidence of GLA IVS4+919 G>A mutation, the underlying mechanisms that contribute to development of FC in such mutation type is also poorly understood. We therefore sought to develop a relevant human *in vitro* system that could be used for investigating the pathogenesis of FC and the development of its therapeutic strategies [48], especially for patients with IVS4+919 G>A mutation. In the present study, we generated the cardiomyocytes (CMs) from patient-specific iPSCs from FC patients carrying IVS4+919 G>A mutation (Figure 1) to investigate the potential biomarkers for assessing the progression of Fabry-associated cardiomyocyte hypertrophy. The established patient-specific iPSC-FC-CMs not only expressed typical cardiac markers, such as TNNT2 and MYL2, but also recapitulated FC characters including the IVS4+919 G>A mutation and lysosomal Gb3 accumulation, decreased GLA enzyme activity and cardiomyocyte hypertrophy (Figure 2), indicating such cells could be used as a feasible experimental platform to study the cardiac functions and signaling in FC. With respective to the results of IPA (Figure 4), IL-18 has been considered to play an important role in progression of hypertrophic cardiomyopathy. Serum samples and biopsied myocardium samples from patients with late-onset FC further confirmed that IL-18 expression was upregulated and its secretion was in circulation at high levels, which suggests that the IL-18-related pathway is activated in FC. In addition, in some Fabry patients with poor response to ERT, IL-18 but not lysoGb3 exhibited better correlation with all detected transthoracic echocardiography parameters. These findings indicated that IL-18 might represent a diagnostic marker in Fabry patients who carried IVS4+919 G>A mutation and have developed LVH.

Accumulating evidences indicate the therapeutic effects of neutralizing anti-IL-18 antibody and IL-18 binding protein (IL-18BP) in various autoimmune diseases, inflammatory bowel disease, sepsis, and acute kidney injury [49]. Because of the deteriorated role of IL-18 in cardiovascular disorders, blockage of the IL-18-induced inflammatory responses has been considered the potential therapeutic strategy for vascular dysfunction and heart failure, e.g. LPS-induced myocardial dysfunction [50]. Notably, neutralization of IL-18 has been suggested to restore the endothelial progenitor cell dysfunction in systemic lupus model [51] and to inhibit neointimal formation in rats with vascular injury [52]. In addition, the ischemia/reperfusion-induced myocardial injury was ameliorated by neutralizing IL-18 with either anti-IL-18 antibody [16] or IL-18BP [53]. Moreover, neutralization of IL-18 has been shown to partially prevent hypoxia-induced cardiac hypertrophy in mice [54]. Collectively, these studies demonstrated the protective effect of IL-18 neutralizing molecules, i.e. anti-IL-18 antibody or IL-18BP, on experimental models of cardiomyopathy. In the present study, we have provided the evidences that IL-18 play a deteriorated role in Fabry-associated cardiomyopathy and LVH, and the combination of ERT and IL-18 neutralization exhibited a remarkable efficacy that reduced cardiomyocyte size and hypertrophy-associated genes in FC-iPSC-CMs. These findings demonstrated that IL-18 is not only a novel diagnostic biomarker but also a potential therapeutic target for the progression of FC. Neutralization of IL-18 plus conventional ERT treatment would be a beneficial synergistic approach for patients with progressive Fabry cardiomyopathy and poor response to ERT treatment, particularly in Fabry patients with IVS4+919 G>A mutation.

The limited efficacy of ERT drugs on myocardial hypertrophy have been reported by various studies. A clinical study demonstrated that long-term treatment of ERT drugs could slow down the progression of left ventricular hypertrophy. It is noticeable that no patients with LVH at treatment onset exhibited a decrease in left ventricular mass after 10 years of ERT [41]. Our previous study found that the Gb3 deposits in cardiomyocytes are cleared in patient with Fabry disease receiving ERT for more than 3 years but the hypertrophy of cardiomyocyte still presented [42]. These findings suggested that deposit of Gb3 might trigger some self-sustained mechanism(s) to induce left ventricular hypertrophy. Our present study suggested that IL-18 might play a role in one of these processes. Several preclinical studies have reported the effective clearance of microvascular endothelial accumulation of Gb3 in various organs including kidneys, heart and skin (37, 55, 56), whereas Thurberg et al. and Keslová-Veselíková et al. consistently reported the poor histological clearance in the cardiomyocytes isolated from Fabry patients by ERT [56, 57]. Despite these published data of ERT treatment on Gb3 deposit, the ranges of cardiac Gb3 clearance are highly variable from patient to patient. In addition, although the restorative effects of ERT on cardiac functions reported in these preclinical data are likely to be secondary to ERT efficacy on vascular endothelial cells, some controversial data have also been reported. In mouse model of Fabry disease, both non-phosphorylated alpha-galactosidase A produced from moss and phosphorylated alpha-galactosidase A were capable of effective clearance of Gb3 in various tissues, including heart, kidney and liver [34] Alternative explanations for the poor Gb3 clearance in heart tissue have also been proposed. One possible explanation could be the difficulty in the detection of Gb3 reduction at the microscopic levels in cardiomyocytes from the endomyocardial biopsy samples. Another possibility could be that, the clearance of Gb3 in cardiomyocytes will require longer periods of ERT treatment, since that Gb3 deposit in terminally differentiated cardiomyocytes stands for the lifetime of substrate accumulation, [56]. Recent evidence showed *in vitro* drug efficacy that directly cleared lysosomal Gb3 with substrate reduction therapy (SRT) drug via glucosylceramide synthase inhibition [31], in which the drug efficacy did not involve any reduction of the Gb3 deposit in microvascular endothelium. In the present study, our findings have indicated that, administration of Replagal (5 μg/ml) at the onset of cardiac differentiation for 60 days substantially prevented the development of cardiomyocyte hypertrophy during the differentiation course (Figure 8B). However, the same treatment failed to restore the cardiomyocyte hypertrophy at post-induction 40 days, a stage of FC-iPSC-CMs that have exhibited severe phenotype of FC, (Figure 8D). Our data supported that early treatment of ERT drug may possess high potential to ameliorate or prevent Fabry cardiomyopathy and other cardiac co-morbidities in Fabry patients.

The mechanisms underlying the upregulation and elevated secretion of IL-18 in FC-iPSC-CMs were assessed, and one of possible cause was the reduced expression and activity of GLA in Fabry disease. In our GLA knockdown study, we only observed a moderate increase in IL-18 secretion by GLA knockdown at post-induction 30 days, but this secretion was indistinguishable among all groups with any given treatment at post-induction 60 days, a stage fully matured FC-iPSC-CMs that exhibited all FC-specific characteristics. These data suggested that low GLA expression might not be the predominant factor that stimulates the IL-18 secretion. Rather than low GLA content and Gb3 deposit, other factors generated during the long course of Fabry cardiomyopathy may also contribute to the high IL-18 secretion. Chévrier et al. have reported that normal autophagic flux is impaired in Fabry disease [58]. Our previous work also demonstrated that several pro-inflammatory cytokines were also involved in the development of Fabry cardiomyopathy carrying IVS4+919 G>A mutation [38]. The mechanisms that links to the high IL-18 secretion in FC-iPSC-CMs remain not fully understood, and future works will be required to elucidate the mechanisms responsible for the regulation of IL-18 secretion in Fabry cardiomyopathy with IVS4+919 G>A mutation.

In the present study, among the 25 recruited Fabry patients who had developed FC for data acquisition and analysis as well as iPSC studies, only 1 female patient with FC was recruited (1/25; women no.= 4%). The major limitation of this study could be that, the patients were also not randomly selected for participation and may not represent the entire population of Fabry patients carrying GLA IVS4+919 G>A mutation. Actually, asymptomatic women were unlikely to be recruited and assigned for the treatment of Fabry disease and its co-morbidities. Previously, Kampmann et al. have reported a large cross-sectional and long longitudinal study of cardiomyopathy in Anderson-Fabry disease [59]. Although there was a strong correlation exists between age and LVM in both hemi and heterozygotes, the onset for the increase of LVM in males was approximately 10 years earlier than in that in females and the increase rate in male is also greater than that in females. Presumably, the slower and variable progression rate of LVM in females has been attributed to the different distribution of Gb3 in cardiomyocytes comparing to males. As for the mutation type reported in our present study, the causes leading to low recruitment rate of female Fabry patients with cardiomyopathy in Fabry patients with IVS4+919 G>A mutation is uncertain. Further approaches and clinical studies will be required to elucidate whether IL-18 also serves a critical role in the pathogenesis of Fabry cardiomyopathy in female patients.

In conclusion, FC-iPSC-CMs represent a novel *in vitro* model that recapitulates the cardiac phenotype of Fabry patients. Combined with proteomic analysis, FC-iPSC-CMs offer a promising platform for investigating the potential biomarkers of FC. Moreover, using biopsy and serum samples, we have confirmed our findings using FC-iPSC-CMs from our clinical patients. In addition, in our *in vitro* iPSC-CM model, the combination of ERT plus IL-18 neutralization effectively ameliorated and prevented the progression of hypertrophy and cardiomyopathy in hypertrophic FC-iPSC-CMs (Figure 8). Finally, our data have illustrated the important role of IL-18 in Fabry-associated cardiac manifestations in Fabry patient carrying GLA IVS4+919 G>A mutation and identified the IL-18 pathway as a potential therapeutic option that should be further elucidated in animal studies and clinical cases of patients.

## MATERIALS AND METHODS

### Human iPSC generation and cultivation

To generate integration-free iPSCs, cells were nucleofected with 3 μg expression plasmid mixture using Amaxa™ human T Cell Nucleofector™ Kit (Lonza). In each nucleofection, 0.83 μg PCXLE-hOCT3/4-shp53, 0.83 μg PCXLE-hSK, 0.83 μg pCXLE-hUL, and 0.5 μg pCXWB-EBNA1 were used. 3 × 10^6^ cells were nucleofected with Amaxa Nucleofector II using program V-024. Cells were cultured the exactly same way as for reprogramming with lentiviral vector expect that every 10–14 days, freshly thawed inactivated mouse embryonic fibroblasts (MEFs) feeder cells were added into each dish. Undifferentiated iPSCs were maintained on inactivated MEFs (50,000 cells/cm^2^) in human ESC medium (DMEM/F12 (Gibco) supplemented with 20% KnockOut serum replacer (KSR; Invitrogen), 0.1 mM non-essential amino acids (Invitrogen), 1 mM L-glutamine, 0.1 mM ß-mercaptoethanol, 10 ng/ml recombinant human basic fibroblast growth factor (bFGF), and antibiotics (Gibco).

### iPSC-derived cardiomyocytes

Cells were dissociated by Versene (Life technologies), then incubate the plate at 37°C, 5% CO2 and wait for 4 minutes. Aspirate the supernatant, resuspend the cells in mTeSR1 + 5 μM Y27632 and seeded onto Geltrex-coated plates at a density of 3 × 105 cells/cm2. Add mTeSR1 + 5 μM Y27632 medium to each well to make a final volume of 2 ml in each well of the 12-well plate. This time point corresponds to day −4. On day −3, day −2, and day −1, aspirate the medium and replace with 2 ml room temperature mTeSR1 per well of the 12-well plate. At on day 0, the cells were treated with 6 μM CHIR99021 (Selleckchem) in insulin-free RPMI/B27 without-insulin medium (Life Technologies) for 24 hours. The medium was replaced with basal medium for another 2 days. At on day 3, the culture medium was subsequently replaced with 5 μM IWP2 (Tocris) in insulin-free RPMI/B27 without-insulin for 48 hours. At On day 7, the culture medium was changed to RPMI/B27 with containing insulin (Life Technologies), and the culture medium was refreshed thereafter every 3 days.

### Alkaline phosphatase staining

First, removed the culture medium and washed by 1xPBS twice, then cells were fixed with 80% alcohol for at least 2 hours at 4°C. To aspirate the alcohol and infiltrated by double-distilled water and then for 2 to 3 minutes. Remove the double-distilled water and add 100 mM Tris-HCl (pH 8.2–8.5) for 5 min. Remove Tris-HCl and add alkaline phosphatase substrate working solution (Vector) for 1 hour. Colonies stained purple indicated positive alkaline phosphatase activity.

### Reverse transcription-polymerase chain reaction (RT-PCR)

Total RNA was isolated with TRIzol Reagent (Invitrogen) and quantified by spectrophotometry at 260 nm. On a GeneAmpÒ PCR System 9700 thermocycler (Applied Biosystems), 3 μg of each total RNA was reverse- transcribed with SuperScript III (Invitrogen) at 55°C for 1 h into total complementary DNA, which was then used as the template for the subsequent PCR reactions and analysis. The PCR reactions involved an initial denaturation at 94°C for 5 min, followed by 25 or 30 cycles at 94°C for 30 s, exposure to an appropriate annealing temperature (58–62°C) for 30 s, and then a final incubation at 72°C for 45 s. The primers and cycling conditions for real-time RT-PCR were shown in Table 1. Amplified RT- PCR products were then analyzed on 2% agarose gels and visualized using ethidium bromide staining and a camera system (Transilluminator/SPOT; Diagnostic Instruments). The gel images of the RT–PCR products were directly scanned (ONEDscan 1-D Gel Analysis Software; Scanalytic Inc.), and the relative densities were obtained by determining the ratio of the signal intensity to the GAPDH or β-actin.

### Genomic DNA extraction and sequence for identifying the genetic mutation

The genomic DNA extraction from iPSC-WT, iPSC-Fabry, CM-WT and CM-Fabry was follow the manuscript of QIAamp DNA Mini Kit. DNA concentrations were determined using a Nanodrop spectrophotometer (Infinigen, USA). The IVS4+919 G>A mutations was characterized by genomic DNA sequencing. The genomic DNA from various cell lines was performed two different stages of PCR to amplify the mutation site for further sequencing.

### Immunofluorescence staining

Aspirate the medium and wash cells twice with PBS. First, cells were fixed with 1% (vol/vol) paraformaldehyde for 10 minutes then aspirate the paraformaldehyde. Second, fixed with 70% alcohol for 10 minutes at room temperature then aspirate the alcohol. Add 0.1 % NP-40 (Sigma #18896–50 ml) for 20 minutes then wash twice with PBS. To block cells with blocking solution (PBS with 0.3%BSA and 5% serum) for 30 minutes to 1 hour. To aspirate the blocking solution then stain cell with primary antibodies in the blocking solution overnight at 4 C. Cells washed three times in PBS, then stained with secondary antibodies at 1:200 in PBS for 1 h at room temperature. Cells were washed three times in PBS, and nuclei were stained with Hoechst 33342 (Life Technologies) at 1:5000 in PBS for 5 min at room temperature. Prior to imaging, cells were inverted onto a glass coverslip containing one drop of SlowFade Gold antifade reagent (millipore) and sandwiched with another glass coverslip on top. DAPI was used as nuclear stain (blue). Images were obtained using fluorescent microscopy and a digital camera.

### Western blotting

Cells were lysed in RIPA lysis buffer (0.5M Tris-HCl, pH 7.4, 1.5M NaCl, 2.5% deoxycholic acid, 10% NP-40, 10 mM EDTA, protease inhibitor), and the protein lysates were subjected to SDS-PAGE followed by electronblotting onto a PVDF membrane. Membranes were probed with monoclonal antibodies against α-galactosidase A (GeneTex) and β-actin (sigma). Bands were visualized by chemilum inescence detection reagents.

### GLA enzyme activity

Cells were washed twice with PBS. Afterward, cells were lysed in 60μl lysis buffer (N-acetyl-D-galactosamine, 4-methylumbelliferyl-α-D-galactopyranoside (4-MUGal), 0.5% Triton X-100). A 4-methylumbelliferone (4-MU) standard curve ranging from 0.15μM to 5000 μM. 10 μl lysate were added to 50 μl Assay Buffer, containing 6mM 4-MUGal and 117mM N-acetyl-D-galactosamine, and incubated at 37°C for 1 hr. Next, 70 μl glycine-carbonate solution was then added to stop the reaction. At last, using a fluorescence with excitation and emission wavelength of 365 nm and 448 nm.

### Karyotyping

The chromosome number and structure were analyzed following the previous description [33]. Briefly, the iPSCs were treated with Colcemid (Gibco) to arrest cells in metaphase, and then harvested within the hypotonic solution to spread the chromosomes. Following fixation with methanol and acetic acid mixture (3:1), the cells were squashed on the slide and then stained with the Wright staining solution (Sigma-Aldrich). Afterwards, the chromosome G-banding were visualized, photographed, and arranged into the karyogram at the TVGH cytogenetics laboratory.

### Transmission electron microscopy (TEM)

Cells were suspended in 1.2% agarose in 0.1 M phosphate buffer (pH 7.4), and fixed with 2% paraformaldehyde and 2.5% glutaraldehyde at 4°C overnight. Following washing with the phosphate buffer, the samples were post-fixed with 1% OsO_4_ in 0.05 M phosphate buffer at RT for 1 hr. After washing with distilled water, the samples were “en bloc” staining with 0.2% uranyl acetate in 70% EtOH at 4°C overnight. The samples were dehydrated in a serial dilution of ethanol for 10 min each (from 70% to 100% ethanol) and further infiltrated with a 100% ethanol/acetone (1:1) mixture and 100% acetone for 15 min each. Then, the samples were embedded by Spurr’s epoxy resin. Briefly, the samples were infiltrated with 100% acetone/epoxy resin (1:1) and (1:3) mixture for 1 hr each, and then transfer to epoxy resin for continuous infiltration for 24 hr. The epoxy resin was polymerized and solidified at 72°C for 72 hr. Following the polymerization, the resin blocks were trimmed and sliced to 80 nm sections using an ultramicrotome (Leica EM UC7, Vienna, Austria). Thin sections were transferred to 200 mesh copper grids and counterstained with 2.5% uranyl acetate for 30 min and 0.4% lead citrate for 4 min prior to observation with a JEM-2000EXII electron microscope (JEOL USA, Inc., Massachusetts, USA).

### Microarray analysis

Total RNA was extracted from 40 days post-differentiated cardiomyocytes by using TRIzol Reagent (Invitrogen) and then sent to the Microarray core laboratory of TVGH for the following cDNA synthesis, labeling, hybridization, and scanning. The GeneChip^®^ Human Gene array from Affymetrix was used and the data normalization, compare, and analysis were conducted by Affymetrix Power Tools. The differentially expressed genes (≥ 2-folds changes) between control and Fabry sample were isolated. Heat map was visualized by MultiExperiment Viewer (MeV) downloaded from the TM4 microarray software suite (http://www.tm4.org/). The color present the normalized expression value of log2 (fold change), and the red indicates up-regulated genes and the blue indicates the down-regulated genes.

### Toluidine blue staining

Tissue specimens of patients with FC were collected and retrieved from the archives of the Department of Pathology of Taipei Veterans General Hospital. The tissue sections were performed Toluidine blue staining after deparaffinization, rehydration, and wash. Briefly, 0.1% Toluidine blue was used to stain the tissue sections. Following the staining, the sections were washed in distilled water three times, dehydrated quickly through 95% and absolute EtOH, cleared in Xylene, and mounted for analysis.

### Quantitative RT-PCR (qPCR)

Following total RNA isolation and cDNA synthesis, the qPCR analysis using Power SYBR-Green PCR master mix (Applied Biosystems) was performed in LightCycler 480 instrument (Roche Diagnostics GmbH). Melting curve analysis was used to confirm the amplification specificity. β-actin was used as an internal control. The relative gene expression levels were determined in comparison with the samples of Ctrl-iPSC-CM.

### Immunohistochemistry (IHC)

The formalin-fixed and paraffin-embedded tissues were sectioned to 4-μm-thick for IHC analysis of the IL18 expression. Briefly, the tissue sections were deparaffinized with xylene, rehydrated through a series of graded ethanol solutions (100%, 90%, 75%), washed with PBS, and retrieved the antigen with 10 mM citrate buffer (pH 6.0) for 40 min under boiling. The Envision detection system (Dako Cytomation, Glostrup, Denmark) was purchase for IHC staining. Following to the manufacture’s instruction, the sections were treated with peroxidase-blocking solution, blocked non-specific binding with 10% non-immune goat serum, probed with the IL18 primary antibody (abcam; ab68435) at RT overnight, stained with peroxidase/DAB+, counterstained with Mayer’s hematoxylin, and then mounted for analysis.

### Protocol for enzyme replacement therapy (ERT) *in vitro* and preclinical studies

For the *in vitro* ERT treatment, the cardiomyocytes were treated with 5 μg/ml Replagal. The *in vitro* concentrations of Replagal is the theoretical maximal plasma concentrations of agalsidase alpha in infused Fabry patients receiving approved doses [34]. The culture medium containing with Replagal were refreshed every two days. Four days post-treatment, the cells were collected for the following analysis. In addition, the enzyme replacement therapy (ERT) administration in patients with FC was supported by Ministry of Health and Welfare in Taiwan based on Gb3 accumulation in cardiac biopsy, and executed by intravenously administration of Agalsidase alfa (Replagal^®^ 0.2 mg/kg) or agalsidase beta (Fabrazyme^®^ 1.0 mg/kg) at a two-week interval for an average of 15.5 months (median; range 6–24). Echocardiography and CMR were used to validate the ERT efficacy and prognosis.

### Statistical analysis

For the human subject data, the variables are presented as the mean ± standard deviation and compared with Student’s *t-test*. A paired *t-test* was used to evaluate ERT efficacy. We used a linear regression model to explore the associations between the changes in the levels of the biomarkers and left ventricular mass index (LVMI) before and after ERT. The statistical analyses were performed with SPSS software version 13.0 (SPSS, Inc., Chicago, IL, USA). The results were considered to be significant at *P* < 0.05. A more detailed description of the methods used in this study can be found in the Online Appendix.

## SUPPLEMENTARY MATERIALS FIGURE


