# Supplementary material for: Interleukin-18 deteriorates Fabry cardiomyopathy and contributes to the development of left ventricular hypertrophy in Fabry patients with GLA IVS4+919 G>A mutation
Source: Oncotarget. 2016 Nov 24;7(52):87161–79. doi: 10.18632/oncotarget.13552 (PMC5349979; doi:10.18632/oncotarget.13552)
Supplement: Supplementary file 1 [file oncotarget-07-87161-s001.pdf]

Interleukin-18 deteriorates Fabry cardiomyopathy and contributes to the development of left ventricular hypertrophy in Fabry patients with GLA IVS4+919 G>A mutation

Supplementary Materials

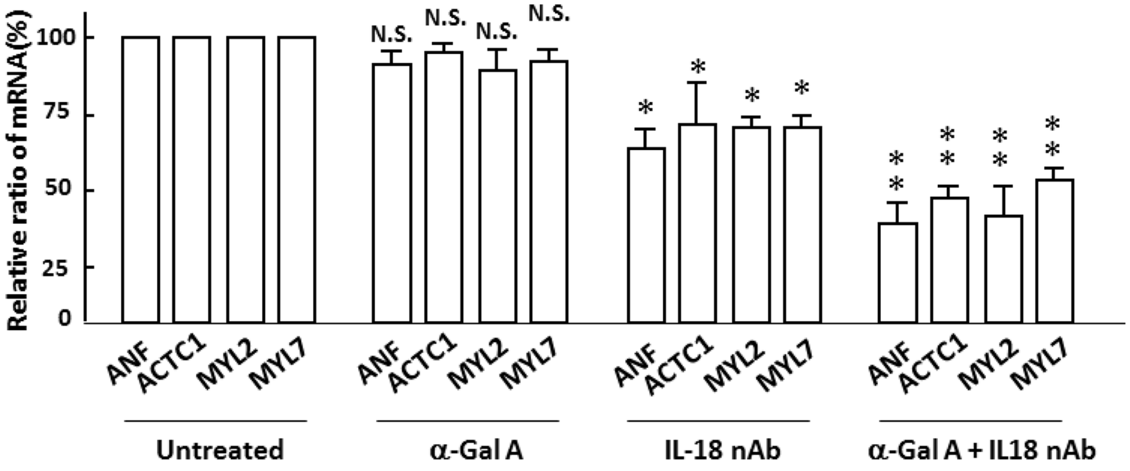

Supplementary Figure S1: Comparison of hypertrophy-associated gene expression patterns in FC-iPSC-CMs carrying IVS4+919 G>A mutation and receiving alpha-galactosidase A and/or IL-18 neutralizing antibodies. Quantitative RT-PCR showing the mRNA expression in FC-iPSC-CMs carrying IVS4+919 G>A mutation and treated with alpha-galactosidase A alone, IL-18 neutralizing antibodies (IL-18 nAb) alone, or the combination of alpha-galactosidase A plus IL-18 nAb. Data shown here is the mean ± SD of three independent experiments, *$P < 0.05$ vs. Untreated.
